# The role of the opponent's head in perception of kick target location in martial arts

**DOI:** 10.3389/fspor.2024.1468209

**Published:** 2024-12-02

**Authors:** M. R. Incognito, T. Watson, G. Weidemann, K. A. Steel

**Affiliations:** ^1^ School of Psychology, Western Sydney University, Sydney, NSW, Australia; ^2^ School of Social Sciences, Western Sydney University, Sydney, NSW, Australia; ^3^ MARCS Institute, Western Sydney University, Sydney, NSW, Australia; ^4^ THRI, Western Sydney University, Sydney, NSW, Australia; ^5^ School of Health Sciences, Western Sydney University, Sydney, NSW, Australia

**Keywords:** accuracy, kick, expertise, face, head, intention, perception

## Abstract

Athletes in Martial Arts must anticipate the target of their opponent's kick or strike to avoid contact. Findings suggest that features, e.g., head and face may play a greater role in predicting opponent action intent compared to global movement information, however little research has explored the role of the head in action anticipation. The aim of this study was to examine the role of the head in predicting the target of a kicking action in martial arts. *N* = 76 volunteer participants (*n* = 32 athletes > 5 years of experience, *n* = 36 non-athletes with no experience) were asked to watch a series of video clips depicting various kicking techniques with differing levels of spatial occlusion of the head. These clips were also temporally occluded compelling participants to predict the landing target of each kick (i.e., head or chest). The hypothesis of the current study is that athletes would be more accurate than non-athletes, but there was no significant effect of expertise on accuracy. Both athletes and non-athletes performed well above chance level performance. Head occlusion did not significantly influence performance and did not interact with expertise, suggesting head and face information did not play a role in predicting opponent action intent. Across participants the landing target of the roundhouse kick was identified with greater accuracy than the front or the back kick. Additionally, participants identified kicks from the rear leg with greater accuracy than the front leg. These findings have significant implications for combat sports where athletes are required to anticipate the action intent of their opponent to formulate an effective defensive response.

## Introduction

Combat sports are dynamic and fast paced, requiring athletes to predict the movement intentions of their opponent based on body movement cues. Fighting stance, head movement and pace of movement are just some of the visual cues athletes use to anticipate their opponent's next attack and inform an appropriate defensive response. Elite performance in fighting sports of Martial Arts (e.g., King Fu, Karate, Judo, Jiujitsu) is dependent on an athlete's perceptual performance, particularly given the constraints presented by the size of the competition space which forces fighters to always remain close to one another (1).

Perceptual-motor expertise gained through task-specific training and effective practice enhances perceptual performance on similar tasks (2, 3). Biological motion research broadly explores the mechanisms underlying perceptual-motor expertise and the contexts where skilled perceptual ability can elicit ideal responses and improve performance (4). This is most relevant to the current study which examines the perceptual capacity of individuals to use biological motion cues to predict attack location in a competitive sporting context.

Research demonstrates that humans can extract internal state and intention information such as fear, emotional states, gender, sexual orientation, and deceit (5–10) from human movement, at above chance levels, depending on the tasks and type of visual display. Techniques that occlude or manipulate movement information and examine the impact this has on perceptual performance, identify the value of certain biological motion cues (11). For example, spatial occlusion of the trunk and attacking arm hindered expert fencers, suggesting they rely on these cues for perceptual performance (12). However, occluding various parts of the body had no impact on perceptual performance amongst Taekwondo athletes, suggesting biological motion perception has the potential to be maintained even when part of the stimulus is visually obstructed (13). This suggests that individuals with perceptual-motor expertise may have developed the capacity to use specific cues learned through experience. That is, they do not need to perceive all details of the stimulus to excel in perceptual performance (14–16).

Disparities in perceptual performance have been found between athlete and novice groups across a variety of ball sports (17–20), indicating that athletes demonstrate a heightened level of perceptual performance by utilizing kinematic cues (21–23). This ability is essential in combat sports such as Martial Arts, where athletes must recognize offensive movements and formulate an appropriate defensive response with speed and accuracy (11). Across a range of combat sports extensive task specific perceptual-motor practice allows Martial Artists to accurately anticipate future movements based on kinematic cues (11, 12, 24–29). However, reaction time findings have yielded mixed results, indicating that athletes perform slower than non-athletes on perceptual tasks (30, 31) or the same (32–35).

Another consideration is the visual strategies used by martial artists to increase intent accuracy of an opponent. This generally consists of individuals scanning along the vertical axis of their opponent's body, shifting their visual gaze between the head and trunk, thus allowing accurate anticipation of future movement. This visual strategy, in which visual gaze is fixed on the most central features of the body, allows for the detection of kinematic changes more efficiently using peripheral vision, thus producing a superior level of perceptual performance (11). Conversely, novices tend to fixate their gaze on the peripheral features such as the upper and lower limbs (12, 24, 26, 28, 36). Therefore, the visual focus combat athletes place on their opponent's chest and head area would seem to play a key role in their performance success. Despite this research, it is unclear what specific information athletes extract from the upper body to guide their performance in combat sports.

The role the head and chest play in guiding perceptual performance is a matter of ongoing debate. The head is the most central and stable part of the body in combat sports and can serve as a fixation point, allowing for efficient use of both central and peripheral vision in detecting movement (37, 38). However, it is possible that facial information provides anticipatory cues given the important survival functions they play in conveying social information related to emotion and intention (39, 40). Facial expressions are perceived in the same automatic manner as movement (41, 42) and have been shown to play a role in accurately predicting sporting outcomes in baseball (43) and Taekwondo (44). However, Prigent et al. (45) examined this effect in basketball and found that facial expressions were used in effort-judgements but not in action-anticipation, with a larger emphasis placed on body movement for predicting the landing location of thrown balls. It is possible that athletes may question their truthfulness in providing accurate anticipatory cues due to the ability to manipulate facial expressions to hide information (46).

In combat sports, anticipatory cues may be extracted from several sources including redistribution of weight, and elevation of centre of mass (2), knee (47), and punch or kick (48). Facial expressions may also aid perceptual performance (49), yet only one study has examined the face and head as a source of perceptual information previously. In the Martial Arts discipline of Petri et al. (23) found that facial information had no effect on perceptual performance, indicating they provide no useful anticipatory cues. Furthermore, the authors suggest that the removal of facial information encouraged athletes to attend to other, more relevant kinematic cues including the head and trunk. Consistent with this, reaction time increased in the presence of facial expressions, potentially due to the additional time required to process facial expressions. However, research specific to combat sports remains limited with no research examining whether perceptual-motor expertise influences the extent to which facial information or other kinematic cues are used to anticipate attack intent.

The purpose of the current study was to examine what visual information Martial Artists and novices utilize during perceptual performance based current research evidence related to the visual cues attained from the head and face and used in movement anticipation. Specifically, we aimed to examine whether the role of the head and face role is limited to a fixation point or whether facial information provides important anticipatory cues used to predict movement outcomes. In line with previous findings, we hypothesized that athletes would make more accurate predictions, and with shorter reaction times than novices across all conditions of spatial occlusion. Additionally, we hypothesized that athlete performance would be hindered by the presence of facial expressions but impeded by the complete removal of the head. We expected to find no differences among non-athletes across spatial occlusion conditions.

## Methods

### Participants

Thirty-three active Martial Arts athletes (males = 20, females = 13; mean_age_ = 34.4 ± 15.6, range_age_ = 18–71) with more than 5 years of experience, forty-four non-athletes (males = 9, females = 34, other = 1; mean_age_ = 23.5 ± 6.4, range_age_ = 18–51) with no martial arts experience volunteered to participate in this study and provided a complete data set. Participants in the athlete group had at least 5 years of training experience (mean years of training = 15.7 ± 8.7) across a range of styles of Martial Arts including Karate, Taekwondo, Kickboxing, Mixed Martial Arts, Muay Thai, and Kung Fu. Non-athletes were recruited from first year psychology students via an online recruitment system and received course credit for participation. Eighty-eight participants provided informed consent in accordance with the ethical procedures approved by the University Human Ethics Committee and completed the online task, but eleven athletes were excluded as they had less 5 years martial arts experience, and a further nine participants (one athlete and eight non-athletes) were excluded due to high levels (>30%) of responses prior to stimulus presentation, indicating that they were not performing the task as instructed [Approval number: H14383].

### Materials

**Figure 1:**
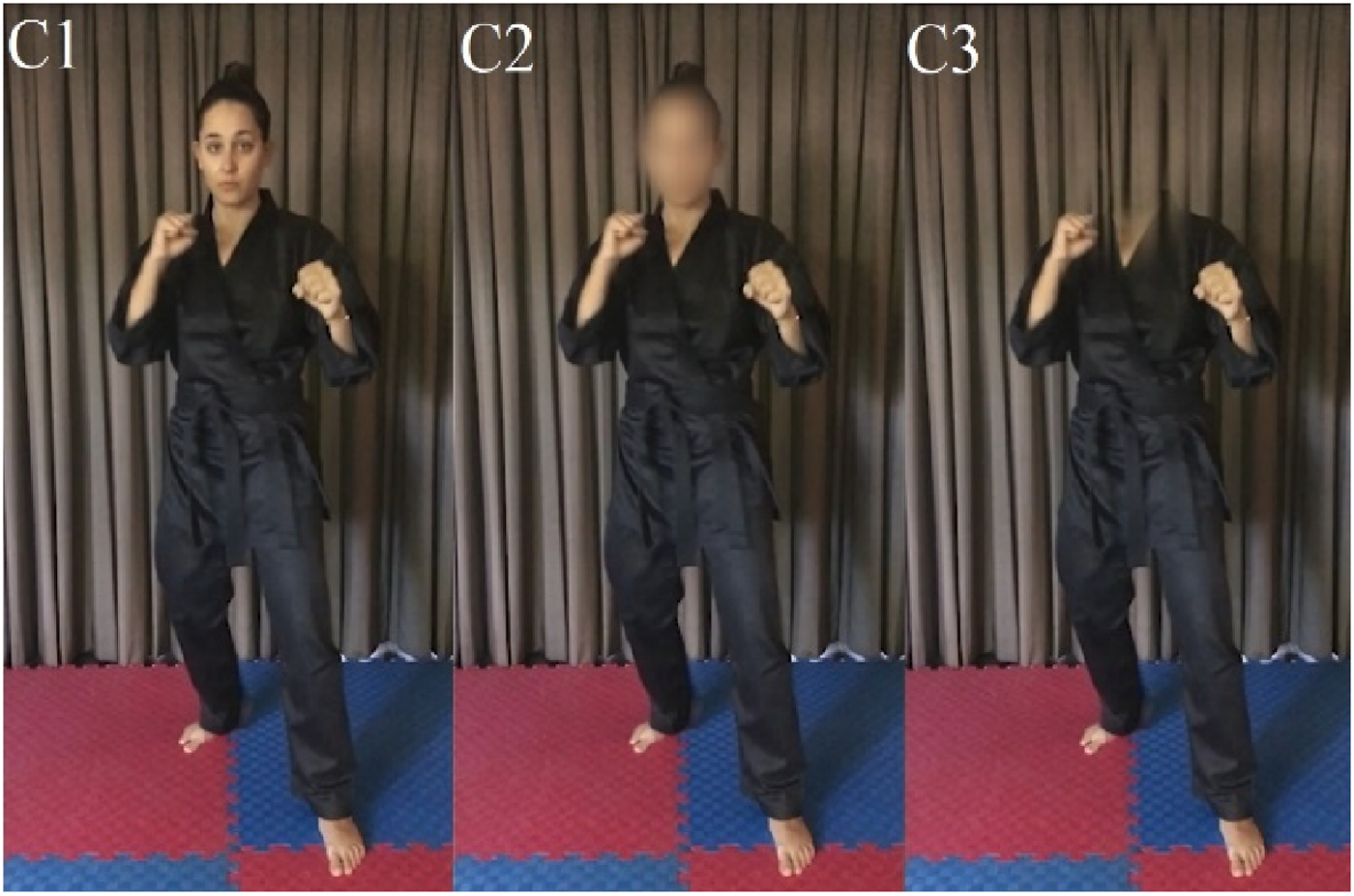
Head and face visibility manipulation. Spatial occlusion conditions. C1 (condition 1): normal; C2 (condition 2): face blurred; C3 (condition 3): head removed.

A spatial occlusion technique was used to manipulate head and face visibility and directly compare the effect this would have on perceptual performance between athletes and non-athletes. Specifically, a video-based task was built and hosted using the online data collection platform Gorilla.sc, and participants were instructed to complete the task on any laptop or desktop computer with a minimum screen size of 14 inches. The video-stimuli used in the task displayed a senior female Martial Artist demonstrating one of three fundamental kicking techniques (front kick, roundhouse kick, back kick) from the first-person perspective, using either the front or rear leg and aimed at either head or chest level. Each stimulus clip began with a freeze frame lasting 3,000 ms, followed by initiation of the technique. The clip was occluded just prior to its completion so the landing location was not visible. Additionally, visibility of the athlete's head and facial expressions were manipulated using the Adobe Premier PRO (version 9.0.0, Adobe Systems). The face was either unmanipulated (C1), blurred (C2) or the head was erased completely (C3) (Figure 1).

### Procedure

The session began with participants completing a short questionnaire that gathered data on age, gender, and level of martial arts experience, which was followed by the video-based task. Prior to commencing the video-based task, participants were told that they would observe a randomized series of clips which depicted a martial artist performing a variety of kicking techniques. After viewing each clip participants were required to indicate whether they anticipated that the athlete's kick was aimed at a hypothetical opponent's chest by pressing the “c” key, or at the head by pressing the “h” key of the computer keyboard. Participants were also given a practice trial prior to starting the test sequence to ensure they were familiar and comfortable with the task requirements. Each video-stimulus was presented at all levels of manipulation twice; 3 head cue manipulations × 3 kick types × 2 kick landing locations × 2 legs × 2 repetitions. The seventy-two trials were presented in randomized order to avoid predictability. The outcome variables were proportion of correct responses (Accuracy) and time taken to respond on correct response (Reaction Time) after the start of the video playback. Any responses made prior to 3,000 ms were made before the onset of biological motion.

## Results

Of the participants who provided a complete data set (*n* = 88), nine participants were excluded from the final analysis as more than 30% of their responses were less than 3,500 ms, indicating that they did not complete the task as instructed leaving (*n* = 79). Thirty-two participants were athletes (>5 years' experience), 36 novices (no experience), and eleven had martial arts experience but less than 5-years' experience. Analysis of athletes and non-athletes was conducted on the data of 68 participants.

To analyze the data, we created a linear mixed-effects model of accuracy and reaction time data in RStudio (version 2023.12.0) using the Lme4 package. We used the linear mixed effects model to analyze the data in preference to an ANOVA due to the complex within- and between-subject nature of the experimental design. The linear mixed effects model is better able to estimate the variance due to subject and condition and to account for any correlated errors in the data, leading to a more accurate estimate of error. The linear mixed effects models included participant group (athlete and non-athlete), head visibility condition (visible, head blurred, head absent), kick type (front, roundhouse, back), kick landing location (head, chest) and leg (front, rear) as fixed effects. A random intercept was included for participants, and random slopes for kick type, kick landing location and leg for the model of accuracy, as this was determined to be the optimal fit. A random intercept for participants, but no random slopes for the other factors, was included in the model of correct reaction-times, as the more complex model did not converge. The effect size of the predictor variable on the outcome variable was assessed using Cohen's *f*^2^, by comparing the full model and the reduced model with the predictor variable removed. Satterthwaite's method was used to determine degrees of freedom and significance of fixed effects and interactions (50) using the afex package. Simulations using this method of significance estimation produced acceptable Type I error rates for small samples (51). As there is no simple way to assess power with a linear mixed effects model, Monte Carlo simulations with the model used have been suggested as the most appropriate way to estimate power. Consequently, we ran simulations using the linear mixed effects model of accuracy with the SIMR package. These simulations showed that there was 76% power to detect a fixed effect of group of 0.2 using the linear mixed effects model employed for accuracy.

**Figure 2:**
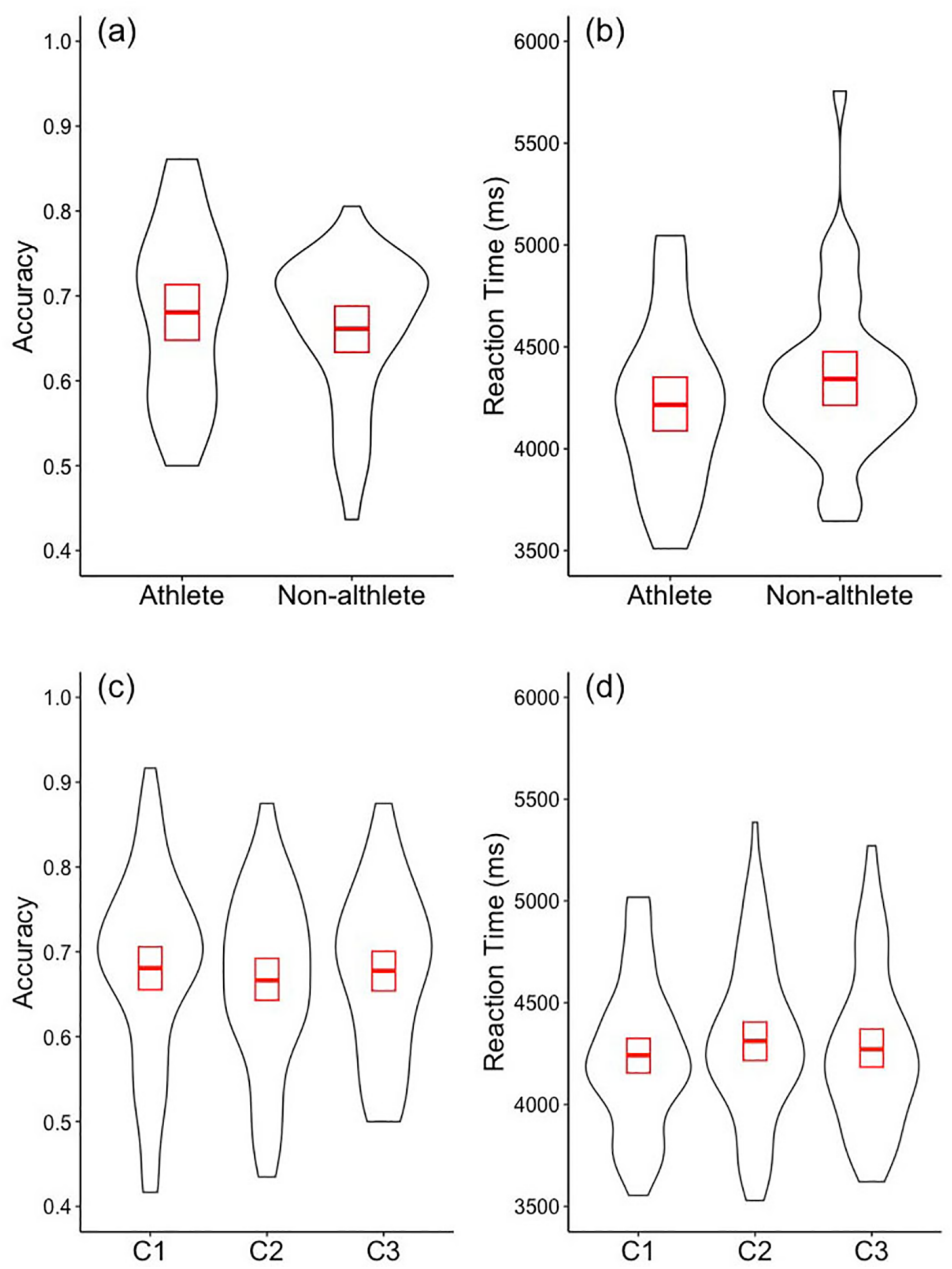
Athlete and non-athlete performance and performance across conditions. Violin plots of the accuracy **(a,c)** and the correct reaction times **(b,d)** of the athlete and the non-athlete participants **(a,b)** and across the three visibility conditions **(c,d)** in determining the target location of the kick. The red rectangles show the 95% confidence intervals, and the red middle line shows the group mean.

Contrary to our hypothesis, athletes (mean = 0.68, sd = 0.10) in our sample were not more accurate than non-athletes (mean = 0.66, sd = 0.09) in determining the target location of the kick, as evidenced by the absence of a main effect of athlete grouping on accuracy, *F* < 1 (Figure 2a). Nonetheless, both athlete and non-athlete participants were above chance in correctly determining the target location [*t*_(31)_ = 9.85, *p* < 0.001; *t*_(35)_ = 11.46, *p* < 0.001, respectively]. Also, contrary to our hypotheses, there was no evidence that athletes (mean = 4,215, sd = 409) were significantly faster than non-athletes (mean = 4,342, sd = 409) in making correct judgements about the target location, with the main effect of athlete grouping failing to reach the declared level of significance, *F*_(1, 67)_ = 1.38, *p* = 0.24 (Figure 2b). Due to the differences in gender balance between the athlete and non-athlete participants we assessed whether there was any evidence of effect of gender on accuracy or reactions times. There is no evidence in the data from the current study of a significant difference in accuracy between male and female participants (male accuracy = 0.686, female accuracy = 0.659, Welch two sample *t*_(45.31)_ = 1.103, *p* = 0.2757, 95% CI = (−0.076 0.022); or of a significant difference in reaction time [male RT = 4,252.284, female RT = 4,305.652, Welch two sample *t*_(57.42)_ = 0.525, *p* = 0.601].

Spatial occlusion did not significantly influence target location accuracy (C1 mean = 0.68, sd = 0.11, C2 mean = 0.66, sd = 0.11, and C3 = 0.66, sd = 0.11) when assessed across all participants, *F*_(2, 4,492)_ = 1.145, *p* = 0.32 (Figure 2c). Furthermore, contrary to our hypotheses, there was no evidence of an interaction between athlete grouping and spatial occlusion on accuracy, *F* < 1. Similarly, across all participants, there was no evidence of differences in correct reaction times as a function of spatial occlusion (C1 mean = 4,229, sd = 374, C2 mean = 4,299. sd = 410, C3 mean = 4,311, sd = 577) *F*_(2, 3,099)_ = 1.463, *p* = 0.23 (Figure 2d), and there was no evidence of an interaction between athlete grouping and spatial occlusion, *F* < 1. However, this result is consistent with the prediction that there would be no differences across spatial occlusion conditions among non-athletes.

**Figure 3:**
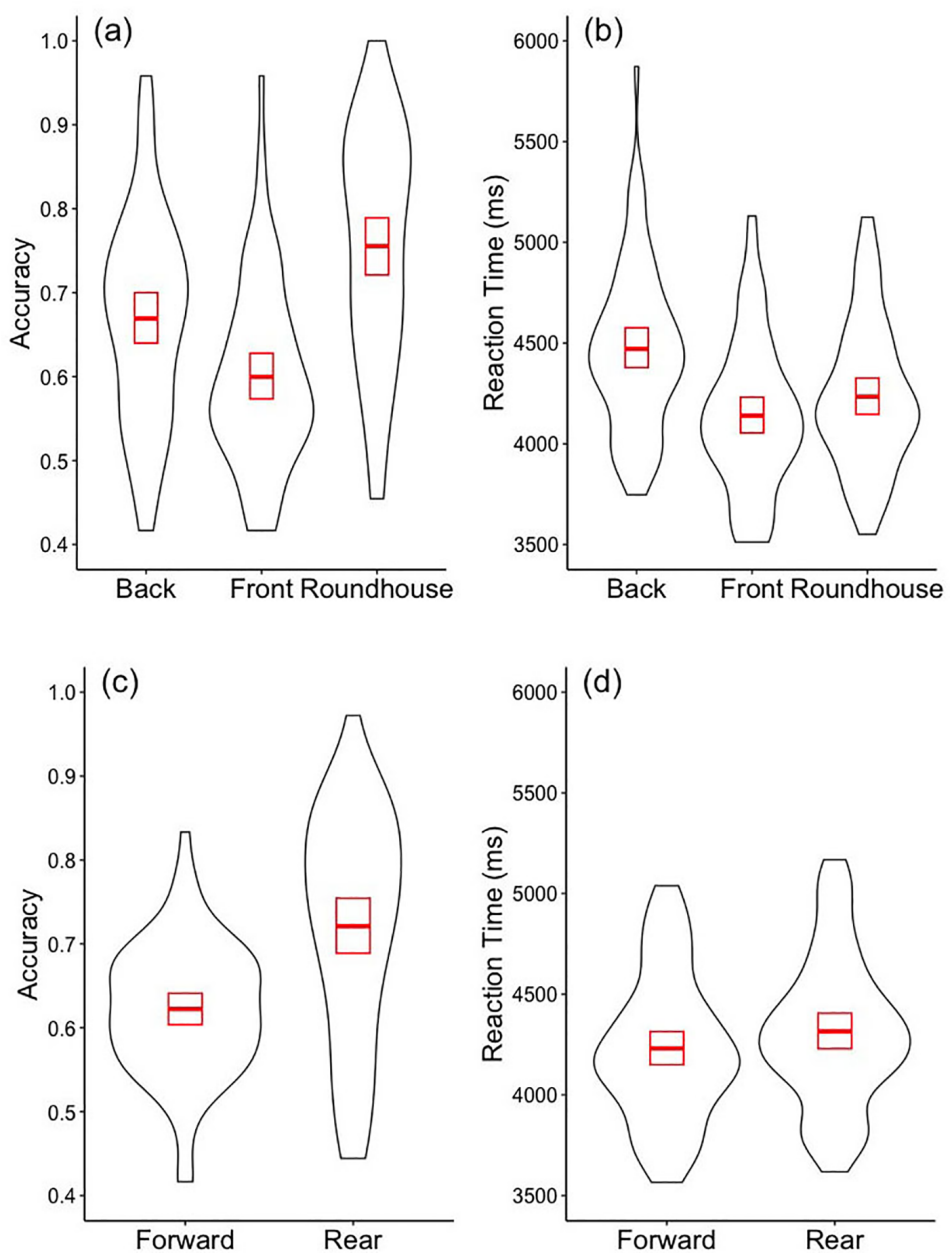
Performance for different kick types and Leg. Violin plots of the accuracy **(a,c)** and the correct reaction times **(b,d)**, across all participants in determining the target location, for the Back, Front and Roundhouse kick types **(a,b)** and for the Forward and Rear leg **(c,d)**. The red rectangles show the 95% confidence intervals, and the red middle line shows the mean for each condition.

Across both athlete and non-athlete participants there were differences in accuracy and correct reaction times for the different kick types, and as a function of which leg was used to perform the kick. Figure 3 shows both accuracy and correct reaction times for the various kick types and for the forward and rear leg. From this figure it is apparent that participants were more accurate in assessing the target location of the kick for the roundhouse (mean = 0.75, sd = 0.15), than for the back (mean = 0.66, sd = 0.13), and the least accurate for the front kick (mean = 0.60, sd = 0.11), evidenced by a main effect of kick type, *F*_(2, 38)_ = 38.59, *p* < 0.001, Cohen's *f*^2^ = 0.21, there was no evidence that this comparison interacted with athlete grouping *F* < 1. Pairwise comparisons between the three different kick types confirmed that accuracy was greater for roundhouse than for front [*t*_(67)_ = 7.52, *p* < 0.001] and for back [*t*_(67)_ = 5.20, *p* < 0.001], and that accuracy for the back was greater than for the front kick [*t*_(67)_ = 3.41, *p* = 0.001]. Reaction times were faster for the roundhouse (mean = 4,261, sd = 615) and the front kick (mean = 4,119, sd = 386) than the back kick (mean = 4,471, sd = 407), which was confirmed by a main effect of kick type, *F*_(2,3,108)_ = 26.19, *p* < 0.001, Cohen's *f*^2^ = 0.03, the interaction between kick type and athlete grouping approached but failed to reach the declared level of significance, *F*_(2,3,108)_ = 2.50, *p* = 0.08. Pairwise comparisons between the three different kick types confirmed that responding was slower for the back than the front [*t*_(67)_ = 10.88, *p* < 0.001] and for the roundhouse kick [*t*_(67)_ = 5.003, *p* < 0.001] but there was no evidence for a significant difference for the front and roundhouse kicks when controlling for multiple comparisons [*t*_(67)_ = 2.11, *p* = 0.038]. From Figure 3 it is also apparent that participants were more accurate in assessing the target location of the rear leg (mean = 0.72, sd = 0.13) than the front leg kicks (mean = 0.62, sd = 0.08), which was confirmed by a main effect of leg position *F*_(1,67.5)_ = 47.39, *p* < 0.001, Cohen's *f*^2^ = 0.07, there was no evidence that this interacted with athlete grouping, *F* < 1. The greater accuracy for the rear leg kicks is associated with a slower reaction time (Rear mean = 4,358, sd = 503, Forward mean = 4,195, sd = 393) which was also confirmed by a main effect of leg position, *F*_(1,3,106)_ = 16.04, *p* < 0.001, Cohen's *f*^2^ = 0.02, but there was no evidence for an interaction with athlete grouping, *F* < 1.

## Discussion

The purpose of this study was to examine the role of the head of an opposing individual when predicting the action intent of kicking techniques in Martial Arts. We hypothesized that athletes would predict the landing location of kicks at different levels of spatial occlusion faster and more accurately than novices. However, data analysis indicates that athletes were no more accurate and did not exhibit faster reaction times than novices, indicating there was no effect of expertise on predicting action intent. This is contrary to movement perception findings which indicate an effect of expertise on action intent accuracy in the sporting field especially balls sports (17–20, 52, 53), and more specifically in the field of combat sports (11, 12, 24–29). We note that there were differences in the gender balance between the athlete and non-athlete groups but there was no evidence that participant gender influenced accuracy or reaction times. Our findings contribute to the mixed results found when exploring the effect of expertise on reaction time in combat sports (30, 32–35, 54–57).

Data analysis showed no significant differences in reaction time or accuracy of action intent estimation between participants across different levels of spatial occlusion of the head. This suggests that head information and more specifically facial information may not always play a critical role in intention prediction in combat sports. This is contrary neurophysiological research using preferential looking paradigms which demonstrates an attentional bias to the human body and face, that emerges early in development and suggests that biological motion perception is hardwired (58, 59). Moreover, some research examining eye movement in combat sports shows that a large visual focus is placed on the head of an athlete's opponent. It is unclear however, whether this is for the purpose of gaze stabilisation (37) or because useful information can be extracted from facial expressions or gaze (49). Because there was no effect of spatial occlusion on perceptual performance, this indicates that kinematic information is extracted from global body information rather than from the head. This aligns with early point-light studies in which masking of body form had no effect on an individual's ability to recognize biological motion (14, 16, 60–62), and where perceptual-motor expertise did not play a role in the global processing of information. Like our findings, research has shown that individuals do not need to perceive all details of a stimulus to perceive action intent, therefore suggesting a global approach to processing of kinematic information (14–16, 23).

Our findings also challenge the idea that perceptual-motor expertise improves an individual's ability to perceive task-specific action intent, with expertise not having a statistically significant effect on accuracy or reaction times. This may be attributed to the study design where we tested recognition, rather than response selection and execution. It is feasible that prioritization of and an ability to recognize biological motion intent is innate, with refinement development from a young age due to the importance socially and for survival (63–65), while perceptual-motor expertise is acquired with experience and used for response selection and execution. For example, Kuhlmeier et al. (63) found that the ability to distinguish non-biological motion from biological motion was already present for 6-month-old infants. This is corroborated by Simion et al. (64) who demonstrated the ability of 6-month-olds to distinguish biological motion attributes including the direction a stimulus moves. Yet the recognition of biological agents does not translate to being able to walk. Infants must first develop muscular strength and neural coordination to maintain head stability, sitting posture and eventually standing posture, only then can they perform skills that they recognize in others (66). This may remain true for adults perceiving biological motion in combat sport contexts whereby the task to identify or recognize threat is hardwired but this does not equate to accurate selection and execution of response. Consequently, future research could examine the differences between athletes and novices in action-response performance.

Different kick types provide differentially salient movement cues that can be used for the prediction of action intent. And while not the intent of this study an unexpected finding arose regarding the effect of kick type and kicking leg on perceptual performance. Data analysis showed that participants could identify the target of a roundhouse kick with greater accuracy than a front or back kick. Like the front kick, the roundhouse kick involves a motion whereby the kicker must lift their knee towards the landing location before extending the leg. Because the kick takes the shape of an arc, the knee must be lifted in a rounded motion, instead of a straight upward motion like that used for the front kick. The roundhouse kick is also a forward-facing kick meaning it is simpler than the back kick, which involves a backwards turning motion that may aid in concealment of useful kinematic cues which predict landing location. It is possible that the redistribution of weight and change in elevation of centre of mass (2) may account for this ability. Alternatively, as the roundhouse kick takes longer to execute (approx. 260 ms) compared to a punch (200 ms) (24), it affords more time to accurately interpret cues (67), or if a rounded motion is just inherently easier to recognize than a straight upward motion (or both).

Speed is not the only important variable when considering the effectiveness of kick types. In fact, a power-speed trade-off may also occur for different kick types. In Martial Arts, variables such as tempo (speed), as well as rotation of the hips are manipulated in the ring so that an athlete can throw kicking techniques at different levels of speed and power to confuse their opponent, thus decreasing predictability (68). For example, the roundhouse kick is typically used in combat sports as a “power kick” (or a “finisher”), and therefore it may be the case that speed is sacrificed to generate enough force for the kick's intended purpose.

Participants in this study, regardless of expertise, also demonstrated greater intent accuracy when the kick originated with the rear leg compared to the front leg. This may be because the rear leg is further away, and hence takes more time to perform a kicking technique, whereas the front leg is closer to the opponent, taking a shorter amount of time to land. Again, it is possible the speed-power tradeoff is at play here, with kicks on the rear leg typically being power kicks. With the roundhouse kick for example, because the leg has further to travel, the athlete has more room to turn their hips and generate a larger amount of force behind the kick. Whereas the athlete has less range of motion to turn their hips and generate power on the front leg as this is closer to their opponent.

This study is the first to have directly investigated whether the head or face is used in action anticipation in combat sports, calling for further investigation using methodologies which produce more generalizable findings. However, we acknowledge that subsequent studies should include other body segments such as trunk/chest. Overall, however these findings have significant implications for combat sports where athletes must anticipate the movement intentions of their opponent and formulate an appropriate defensive response before the movement is completed. This provides real-world implications in terms of informed evidence-based training interventions in combat sports. The increased salience of certain types of kicks delivered on specific legs can inform training practices and fight strategy to help fighters gain an advantage by utilizing techniques that are hardest to identify and respond to. In addition, this knowledge can inform training programs used to enhance the safety of individuals across many lines of work including police officers, paramedics, security guards and defence personnel, in terms of teaching physical self-defense techniques that are difficult to identify and therefore increase their safety.

The findings also align with global processing of information which posits that biological motion is perceived at a global level and can be maintained even when the stimulus is visually occluded. Because there were no differences in performance between groups this suggests global processing is not dependent on perceptual-motor expertise. However, further research is needed to establish what then separates experts from novices, as it may not necessarily be recognition of movement but what follows, e.g., response selection and execution. Further research may also be needed to establish how we can increase our ability to identify less salient kicks of certain legs, which could then inform interventions aimed at improving movement perception. Lastly, future research should incorporate a broader range of skill-based stimuli, including strikes, punches, and diverse kicking techniques from various martial arts disciplines. This approach will not only diversify the stimulus set and contribute to advancing knowledge and techniques but also facilitate the use of match-based stimuli, thereby enhancing ecological validity by replicating more realistic competitive environments.

## Conclusion

The goal of this research was to investigate the practical applications of the perception of biological motions cues to sports performance, specifically, the role of the head when determining action intention associated with different kick types commonly used in martial arts. The results of this study indicated that head occlusion did not significantly influence performance or interact with expertise. This suggests head and face information did not play a role in predicting opponent action intent. Moreover, across participants the landing target of the roundhouse kick was identified with greater accuracy than the front or the back kick. The difference in accuracy between kick types is likely a result of the increased exposure time afforded by a roundhouse kick. These findings suggest that attention to head and face in Martial Arts does not enhance intent estimation and therefore should not be a significant focus during training sessions.

## Data Availability

The raw data supporting the conclusions of this article will be made available by the authors, without undue reservation.
